# Circulation of new lineages of RSV-A and RSV-B in Kuwait shows high diversity in the N- and O-linked glycosylation sites in the G protein between 2020 and 2022

**DOI:** 10.3389/fcimb.2024.1445115

**Published:** 2024-08-16

**Authors:** Nada Madi, Mohammad Sadeq, Hussain A. Safar, Anfal Al-Adwani, Mariam Al-Turab

**Affiliations:** ^1^ Virology Unit, Department of Microbiology, College of Medicine, Kuwait University, Kuwait City, Kuwait; ^2^ Jaber Al-Ahmad Armed Forces Hospital, Ministry of Health, Kuwait City, Kuwait; ^3^ Research Core Facility and OMICS Research Unit, College of Medicine, Kuwait University, Kuwait City, Kuwait

**Keywords:** respiratory syncytial virus, G protein, nanopore sequencing, Kuwait, glycosylation

## Abstract

The human respiratory syncytial virus (RSV) is a significant health concern, particularly for infants, young children, and the elderly. This virus is known to evolve continuously due to environmental factors and herd immunity. In light of this, our study aimed to analyze the genetic variability of the G protein in RSV-A and RSV-B genotypes in Kuwait from 2020 to 2022. Between January 2020 and September 2022, we collected 490 respiratory samples from hospitalized patients with acute respiratory tract infections. These samples were tested and confirmed positive for RSV using multiplex Real-Time PCR. Subsequently, the samples underwent nucleic acid sequencing using the advanced Nanopore sequencing technology to analyze the full-length G gene. Sequence analysis showed that 64 isolates (76%) were RSV-A, and 20 isolates (24%) were RSV-B. The G genes of RSV-A belonged to genotype GA2.3.5, while all the RSV-B genotypes belonged to GB5.0.5a. New lineages and sub-lineages of RSV-A and RSV-B were detected, indicating the circulation of new strains in Kuwait. Many unique and new amino acid changes, including insertions, were found in the G proteins of Kuwaiti isolates, with the highest variability in the second hypervariable region. An increased number of N and O-linked glycosylation sites were also identified in the G protein, which could speculate to alter the antigenicity of RSV. The identified changes in the G protein of RSV-A and RSV-B genotypes might result from immune pressure and could affect the antigenic characteristics of circulating strains in Kuwait. This could potentially lead to new RSV variants that can evade the immune response. Our in-depth analysis of the G proteins of both RSV-A and RSV-B could aid in the development of more potent treatments and vaccines.

## 1 Introduction

Human respiratory syncytial virus (RSV) is the most frequent virus infecting infants, premature babies, the elderly, and immunocompromised individuals (Hall et al., 2013; Schobel et al., 2016). Globally, infection with RSV resulted in 33 million episodes of RSV-associated acute respiratory infection (ARTI), with 3.6 million hospitalizations and 26,300 in-hospital deaths among children younger < than five years. Moreover, among infants,1.4 million episodes of RSV-associated ARTI were recorded, resulting in 13,300 in-hospital deaths (Li et al., 2022). In the elderly, data estimated 1.5 million episodes of ARTI caused by RSV infection in high-income countries, with approximately 15 % (214,000) of hospital admissions (Shi et al., 2020) RSV belongs to the Paramyxoviridae family. It has negative-sense, single-stranded and non-segmented RNA of approximately 15.4 Kb in size. RSV has three surface glycoproteins: F (fusion protein), G (glycoprotein) and SH (small hydrophobic protein). The F and G proteins are two vital membrane proteins that play critical roles in viral attachment and entry to the host cell. In addition, the two proteins are the major antigens that provoke neutralizing antibody responses (Widjojoatmodjo et al., 2010; Lu et al., 2019). RSV is classified into two main genotypes, RSV-A and RSV-B, based on the genetic heterogenicity of the G protein in which the C-Terminal region of this protein (the second hypervariable region, HVRs) is highly variable due to mutations, and therefore, it has been used for genotyping (Peret et al., 2000) The G protein is a type II, a highly glycosylated membrane protein of 292-319 amino acids (aa), consisting of intracellular cytoplasmic tail (aa 1-37), transmembrane domain (aa-38-66), and the extracellular domain ending at its carboxy-terminus (Gimferrer et al., 2015; Ahmed et al., 2016; Anderson et al., 2021) The extracellular domain is rich in serine and threonine residue, and therefore, it is highly glycosylated with 4-5 N-linked glycans and 30-40 O-linked glycans in this region (McLellan et al., 2013; Anderson et al., 2021)The G protein is a key target for neutralizing antibodies, and the characterization of the G protein of RSV is crucial for developing and improving vaccines against it. This is because the G protein plays a significant role in causing inflammation and disease by affecting the host response. It can also bind to monoclonal antibodies, which can help prevent disease. In addition, *in vitro* studies have shown that the G protein helps bind to CX3CR1 in primary human airway epithelial cells, and using anti-G antibodies effectively neutralizes RSV in these cells. Therefore, using vaccine-induced or passively administered antibodies or antiviral drugs targeting the G protein can reduce virus-induced inflammation and virus replication, ultimately leading to a decrease in disease (Anderson et al., 2021). It’s important to note that although the G protein is crucial, the current RSV vaccine candidates undergoing clinical trials are focused on the RSV F glycoprotein. This glycoprotein is conserved in RSV genotypes A and B, facilitates viral fusion and host-cell entry, and stimulates the production of neutralizing antibodies (Papi et al., 2023).

This study aimed to comprehensively analyze and describe the genetic variability of the G protein in RSV-A and RSV-B genotypes present in Kuwait between 2020 and 2022. We believe that examining the common and unique mutations in the G protein of RSV genotypes circulating in Kuwait, their prevalence, conducting phylogenetic analysis, identifying the glycosylation sites, and predicting the effect of amino acid changes on the G protein epitopes could significantly impact the development of an effective RSV preventative.

## 2 Materials and methods

### 2.1 Study population

Between January 2020 and September 2022, 7,093 respiratory samples were collected from hospitalized patients with respiratory tract infections (RTIs) at Mubarak Al-Kabeer Hospital. The patients had various respiratory tract diseases, such as bronchiolitis, pneumonia, acute respiratory distress syndrome (ARDS), croup, bronchopneumonia, and acute nasopharyngitis. Different respiratory samples were collected from patients, including throat and nasal swabs, nasopharyngeal swabs, nasopharyngeal aspirates/wash, sputum, tracheal aspirates, and bronchoalveolar lavage (BAL). Respiratory samples collected at Mubarak Al-Kabeer Hospital’s Virology Unit were screened for various respiratory viruses, including RSV, using Real-Time multiplex PCR. The assay used can detect a range of viruses such as influenza A, influenza A (H1N1), influenza B, human rhinovirus (HRV), human coronaviruses NL63, 229E, OC43, and HKU1, parainfluenza virus (PIV) types 1-4, human metapneumovirus (HMPV) A/B, bocavirus, respiratory syncytial virus (RSV) A/B, adenovirus (AdV), enterovirus, parechovirus, and mycoplasma pneumonia. Internal control was also included in the screening, using a Fast Track Kit from Fast Track Diagnostics in Luxembourg. The samples were transferred to the Virology Unit, College of Medicine, Kuwait University, on ice and then processed and stored at −70°C till RNA was extracted. The Ministry of Health, Kuwait Ethics Committee, and Research Committee of the College of Medicine approved this study (No: 2019/1203). This study did not require informed consent as it utilized residues of stored samples per national legislation and institutional requirements.

### 2.2 RT-PCR for the G protein gene

Nucleic acid isolation from PCR-confirmed clinical samples was carried out using the Roche MagNA Pure LC system (Roche Diagnostics, Indianapolis, IN, USA), according to the manufacturer. RiboLocl RNase Inhibitor (Thermo Fisher Scientific) was added to the extracted samples to preserve the extracted RNA. As described previously, a one-step RT-PCR kit (QIAGEN, Hilden, Germany) was used to amplify the G gene (Madi et al., 2018).

### 2.3 Sequencing of G protein gene using MinION nanopore technology

The whole RSV genome was amplified using 19 in-house overlapping primers, including primers targeting the open reading frame of the G protein gene of RSV-A and RSV-B genotypes. A One-Step RT-PCR Kit (QIAGEN, Hilden, Germany) synthesized and amplified the cDNA from the extracted RNA according to the manufacturer’s instructions. The amplicons of the G protein gene were used for sequencing using Oxford Nanopore sequencing technology (Oxford Nanopore Technologies, Cambridge, United Kingdom). Briefly, after amplicon cleaning up with AMPure XP beads (Beckman Coulter Diagnostics, California, United States), a ligation sequencing kit (SQK-LSK109) from Oxford Nanopore Technologies (Oxford, United Kingdom) was used to prepare the libraries. DNA ends repairation and end-prepped/dA-tailed were performed using the NEBNext Ultra End Repair/dA-tailing module kits (E7546, New England BioLabs (NEB), Ipswich, MA). Later, Native Barcoding Expansions 1-12 (EXP-NBD104) and 13-24 (EXP-NBD114) were used for native barcode ligation. To the pooled and barcoded DNA, sequencing adapters were added using Adapter Mix and Quick T4 DNA Ligase with Ligation Buffer (NEB). Then, 15 ng of the library (quantified using QUBIT 1X dsDNA HS Assay Kit (Invitrogen, Waltham, United States), was loaded into Oxford Nanopore MinION SpotON Flow Cells FLO-MIN106D, R9.4.1 (Oxford Nanopore Technologies, Oxford, United Kingdom). The FastQ files generated by the Mk1C device were used for analysis.

### 2.4 Phylogenetic analysis of the G protein gene of RSVA and B

The FASTQ reads generated by the MK1C device were processed using Guppy version 3.1.5 and then aligned to either of the RSV reference genomes (NC_038235.1 Human orthopneumovirus Subgroup A and NC_001781.1 Human orthopneumovirus Subgroup B). FASTA consensus files were generated using SAMTOOLS (v1.13) and BCFTOOLS (v1.5). G protein gene sequences were selected and used for multiple sequence alignments with known RSV genotypes using MUSCLE (Multiple Sequence Comparison by Log Expectation) algorithms in MEGA software (MEGA 11 v11.0.13: Molecular Evolutionary Genetics Analysis across computing platforms) (Tamura et al., 2021). The RSV-A and RSV-B reference sequences were downloaded from the National Center for Biotechnology Information (NCBI) database. Genotype assignment and identification according to the G protein gene classification (Goya et al., 2020). were performed using the Nextrain genetic analysis platform (Hadfield et al., 2018). The phylogenetic trees of nucleotide sequences for RSV-A and RSV-B genotypes were constructed using the Maximum Likelihood method under Tamura-Nei/JTT matrix-based models in MEGA11 v11.0.13. The robustness of the trees was assessed with 1,000 replicas. The Interactive Tree of Life (iTOL) v6 software visualized and modified the phylogenetic trees.

### 2.5 Analysis of deduced amino acid sequences and mutations

The amino acid sequences of the G protein gene of RSV-A and B genotypes were predicted with standard genetic code, and the mutations and their frequencies were described for group A and group B concerning their prototype strains using MEGA11 software.

### 2.6 Entropy analysis

Variations in the amino acid of the G protein gene were performed using Shanon entropy in BioEdit (ver 7.2.5). Shanon’s entropy threshold value was 0.2; amino acids with <0.2 were considered conserved sites, while values >0.2 were considered variable sites.

### 2.7 N and O-linked glycosylation sites analysis

Putative N-glycosylation sites in the G protein were predicted using NetNGlyc 1.0 server (threshold ≥0.5), while O glycosylation sites were predicted using NetOGlyc 4.0 server (G-score ≥0.5).

## 3 Results

### 3.1 Patient characteristics

Out of 7,093 respiratory samples, 490 (6.9%) of respiratory samples from hospitalized patients between January 2020 and September 2022 tested positive for RSV using multiplex Real-Time PCR. The patients’ ages ranged from under one year to 97 years, with a median age of less than one year. The male-to-female ratio was 1:1. The majority (69%, n=338) of the samples were from infants under one year old, 16% (n=79) were from children aged 1-5 years, 7% (n=34) were from patients over 60 years old, 5% (n=27) were from patients aged 29-60 years, 2% (n=8) were from patients aged 14-28 years, and only 1% (n=4) were from patients aged 6-13 years.

### 3.2 Differential distribution of RSV-A and RSV-B subtypes

Out of the 490 RSV-positive respiratory samples, 84 high-quality consensus G protein gene sequences were successfully obtained and assembled using Nanopore sequencing technology and were used for downstream analysis. G protein gene sequence analysis revealed 64 (76%) RSV-A genotypes and 20 (24%) RSV-B genotypes. According to Nextstrain platform analysis, RSV-A sequences were divided into two G clades: GA2.3.5 sub-genotype (n=38; 59%) and GA2.3.3 sub-genotype (n=26; 41%). On the other hand, all RSV-B sequences were from the GB5.0.5a sub-genotype. All the 64 study sequences were deposited in the GenBank with the following accession numbers: PP135042-PP135061 for the RSV-A and PP151342-PP151405 for the RSV-B (**Supplementary Table 1**).

### 3.3 Phylogenetic analysis of RSV-A and B G glycoprotein

**Figure 1:**
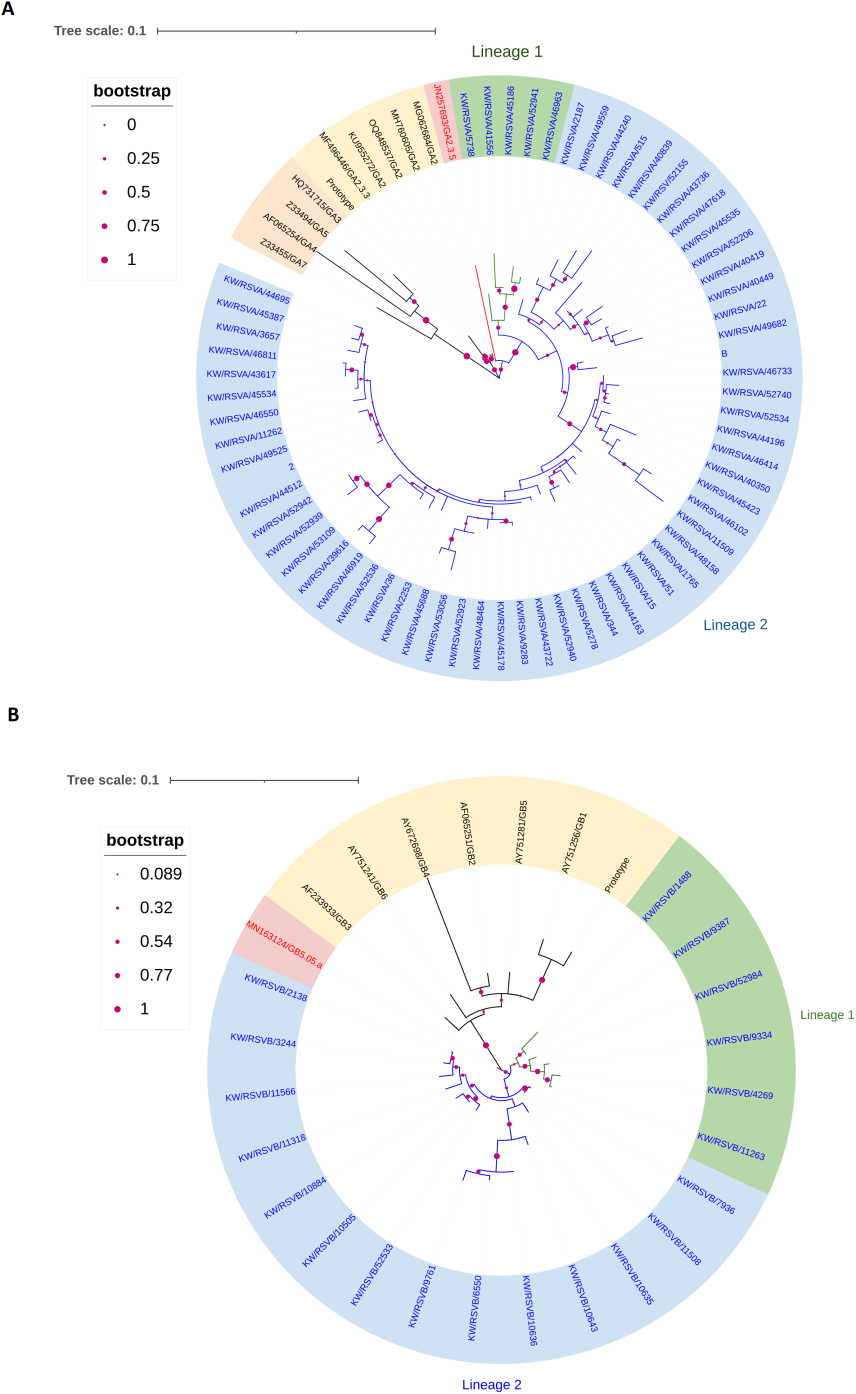
Phylogenetic tree of RSV-A **(A)** and RSV-B **(B)** in Kuwait. The phylogenetic trees G protein gene was constructed using the Maximum Likelihood method and General Time Reversible/JTT matrix-based models. Kuwaiti strains are blue, and reference strains are black. The scale bar indicates the proportion of nucleotide substitutions, and the branch nodes show bootstrap values with marron dotes. **(A)** The phylogenetic tree of RSV-A genotypes (GA2.3.5 sub-type) in Kuwait is divided into two lineages: lineage 1 is shaded in green, and lineage 2 is shaded in blue. **(B)** The phylogenetic tree of the RSV-B genotype (GB5.05.a subtype) in Kuwait is divided into two lineages: lineage 1 is shaded in green, and lineage 2 is shaded in blue.

Phylogenetic analysis of the G protein gene of 64 samples using reference sequences of known G genotypes revealed that all RSV-A genotypes were of GA2.3.5 sub-genotype and clustered with GA2.3.5 reference genotype with bootstrap support of 50%, an average pairwise nucleotide distance of 0.07 and 93% of nucleotide identity. The nucleotide identity between Kuwait RSV-A strains was 97% and was divided into two main lineages supported with a bootstrap of 97% and an average pairwise difference of 0.03. However, lineage 2 was further divided into two sub-lineages with an average pairwise difference of 0.025 (**Figure 1A**). Phylogenetic analysis of the G protein gene of the 20 RSV-B genotypes comprised the GB5.0.5a genotype and formed one lineage clustered with GB5.0.5a reference genotypes with bootstrap support of 99%, an average pairwise nucleotide distance of 0.03, and 97% of nucleotide identity. The nucleotide identity between Kuwait RSV-B strains was 97%, and this lineage was subdivided into two sub-lineages with bootstrap support of 50% and an average pairwise difference of 0.025 (**Figure 1B**).

### 3.4 Entropy analysis

**Figure 2:**
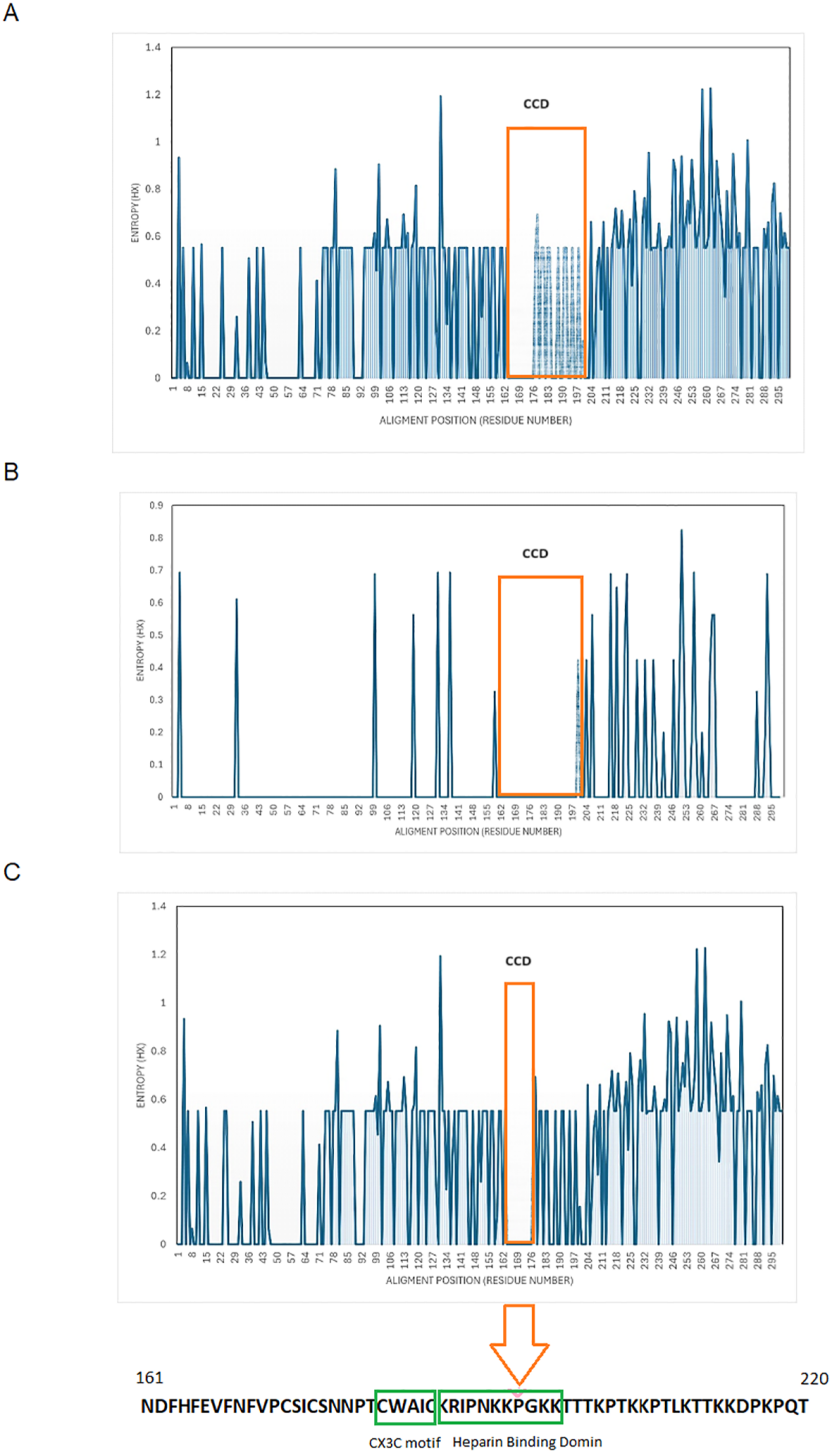
Shanon entropy plots of deduced amino acid sequences of G protein. **(A)** RSV-A, n=64) **(B)** RSV-B, n=20) **(C)** all Kuwaiti strains (N=84) with their respective prototype strains. The threshold value was set at 0.2. Amino acid sites with entropy values <0.2 are considered conserved, and values >0.2 are deemed viable. The 13 aa, aa 164-176, conserved among all strains, is in orange boxes. The Lowes panel shows aa sequences that include CCD, the CX3C motif (aa 182-186), and the HBD (aa 197-198). CCD, central conserved domain.

Shannon entropy analysis of the entire region of G protein was carried out for all the RSV-A and RSV-B genotypes circulated in Kuwait, with relevant prototype strains. The analysis of the RSV-A genotype revealed that the G region is highly variable, with more than 160 variable sites distributed throughout the G protein sequence and having an entropy value of more than 0.5 (**Figure 2A**). Two different amino acids at positions 258 and 262 (second hypervariable region) were the most variable in the RSV-A genotype (entropy value=1.22). On the other hand, the RSV-B genotype’s G protein was less variable, with only 17 variable sites having an entropy value of more than 0.5 (**Figure 2B**). Amino acids at positions 251 and 257 (second hypervariable region) were the most variable in the RSV-B genotypes (entropy value=0.82 and 0.69, respectively). The central relatively conserved domain (CCD) (aa 160-200) in the RSV-A genotype was conserved at aa 162 to 176 (**Figure 2A**), while the CCD in the RSV-B genotype was conserved at aa 162 to 197 (**Figure 2B**); this resulted in a net of 13 aa (aa 164-176) conserved region of CCD among RSV-A and RSV-B genotypes (**Figure 2C**). CCD has a CX3C chemokine motif (aa182-186) and a heparin-binding domain (HBD) at aa 187-198; both regions were conserved among Kuwaiti RSV-A and B genotypes.

### 3.5 Mutations and amino acid changes in G glycoprotein

**Figure 3:**
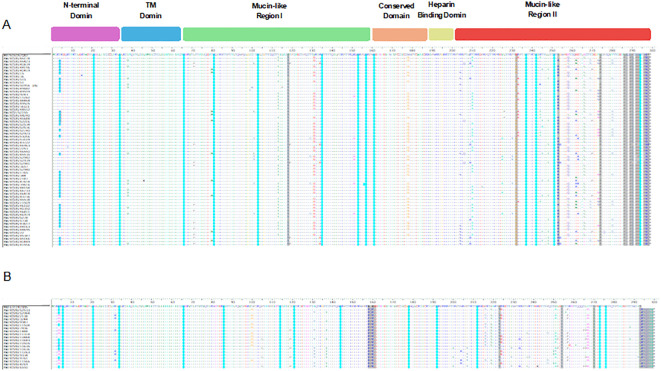
Descriptive alignment of the G protein of RSV-A and B genotypes circulated in Kuwait. **(A)** alignment of RSV-A genotype with MH760605/GA2 prototype strain **(B)** alignment of RSV-B genotype with MN163124/GB5 prototype strain. Dots display identical residues. Stop codons are displayed by asterisks. Molecular markers are indicated in grey shading. The potential N-glycosylation sites (NXT, where X is not proline) are indicated by blue dots. The potential sites for extensive O-glycosylation are not shown.

The diversity of amino acid changes in the G protein of RSV-A and RSV-B was analyzed, and single nucleotide polymorphism (SNP) calling was performed with respect to prototype strains, as shown in **Figure 3**. The number of SNPs in the G protein region indicated a high genetic diversity level of RSV-A and RSV-B genotypes. The rate of coding SNP detected in the G protein gene of the RSV-A genotype was 17% (n=52), while the rate of coding SNP detected in the G protein gene of the RSV-B genotype was (12%) (n=36). The highest SNP in the G protein of RSV-A and B genotypes were in the second highly glycosylated variable mucin-like domain ending in the carboxy-terminals (**Figures 3A, B**). To determine the molecular marker, the fixation percentage of changes in every amino acid position in the G protein in each genotype was calculated and considered a molecular marker if the fixation percentage exceeded 75%. The G protein of the RSV-A genotype contained 12 molecular markers: I118T in the first variable mucin-like domain, while the other 11 (E232G, T253K, L274P, L286P, S289P, P290S, S292P, S293P, T296P, K297R, and the stop codon 297Q located in the second highly glycosylated variable mucin-like domain. G protein of the RSV-B genotype, on the other hand, had 13 molecular markers: 158K (insertion), 159P (insertion), 160K (insertion), and P223T located in the first variable mucin-like domain; I254T, T270I, stop codon 293Q/L, 294K/N (insertion), 295T (insertion), 296Q (insertion), 297S (insertion), 298Y (insertion), and 299A (insertion) located in the second highly glycosylated variable mucin-like domain (**Figure 3B**). Other mutations were detected in lesser percentages in RSV-A and RSV-B genotypes and are presented in **Supplementary Table 2**.

### 3.6 N- and O-linked glycosylation sites

N-X-S/T, where X was not Pro, was identified as the N-glycosylation site. Compared to their prototype strains, N-linked glycosylation sites were predicted for RSV-A and RSV-B genotypes (**Figure 3**). For RSV-A strains, 13 major putative N-glycosylation sites were detected and conserved in all the strains, including the prototype strain. However, the N-glycosylation site N4 was predicted in 63% of RSV-A strains but not in the prototype strain (**Figure 3A**, blue dots). Additionally, 13 major putative N-glycosylation sites were identified for RSV-B strains and their respective prototype, which were conserved in all the strains. However, N-glycosylation site N4 was predicted in 50% of RSV-B strains, including the prototype strain (**Figure 3B**, blue dots). Serine and threonine residues were predicted as potential O-linked glycosylation sites for RSV-A, and 70 sites were intensively distributed in the G protein from aa 64 to aa 291 with a G score range of 0.50-0.99. However, the serine and threonine potential O-linked glycosylation sites in RSV-B were 71 sites distributed in the G protein from aa 70 to aa 290 with a G score of 0.53- 0.98 (data not shown).

### 3.7 Analysis of the G protein second hyper-variable region of RSV-A and B

**Figure 4:**
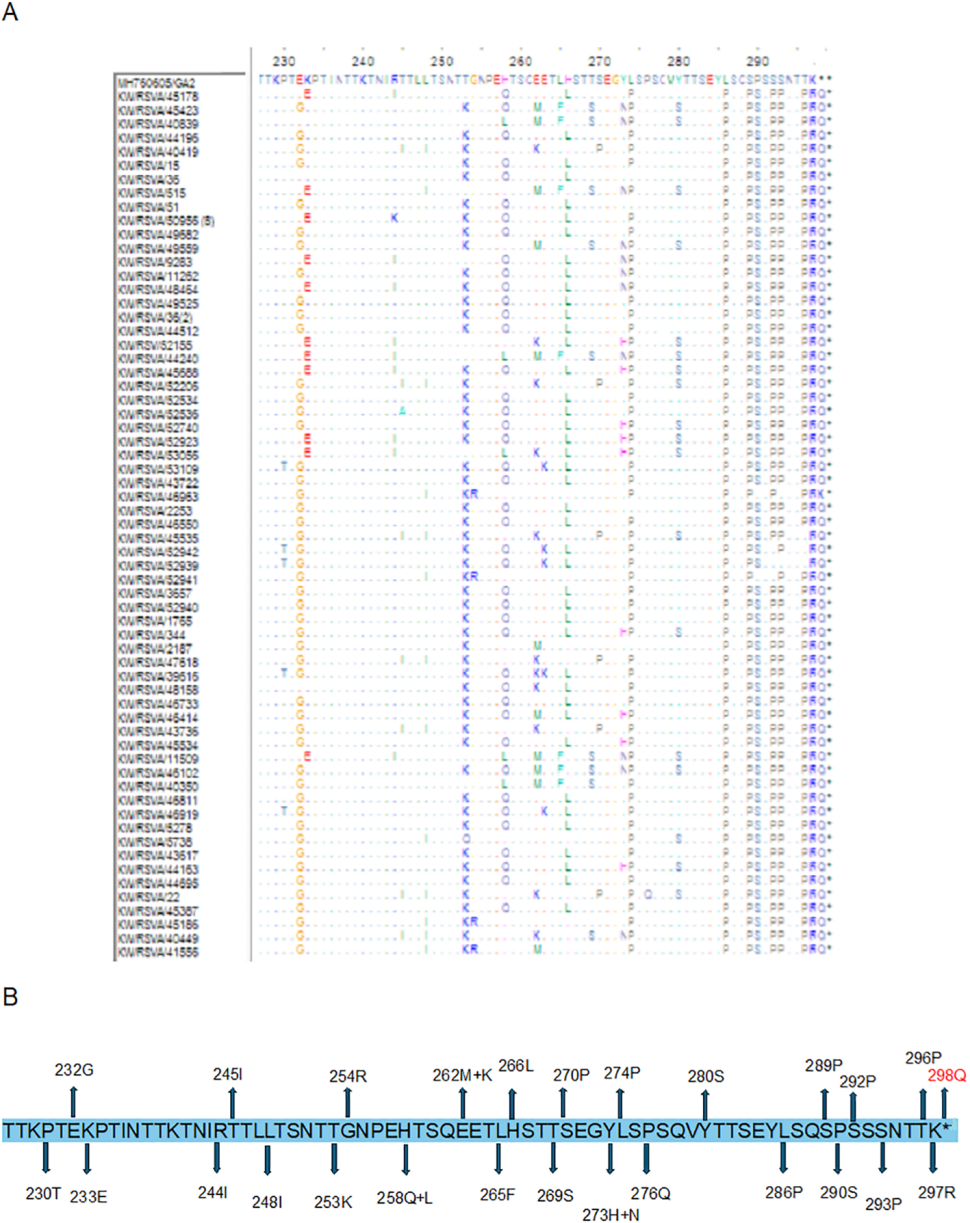
**(A)** Deduced amino acid alignment and mutations in the second hyper-variable region of the G protein (227-298) of RSV-A genotype. The Kuwaiti strains were aligned with the MH760605/GA2 prototype strain. Dots display identical residues. Stop codons are indicated by asterisks. **(B)** mutations in the amino acids of the Kuwaiti strains. An asterisk shows the stop codon in the prototype strain. Amino acid in red is inserted AA in the Kuwaiti strains.

**Figure 5:**
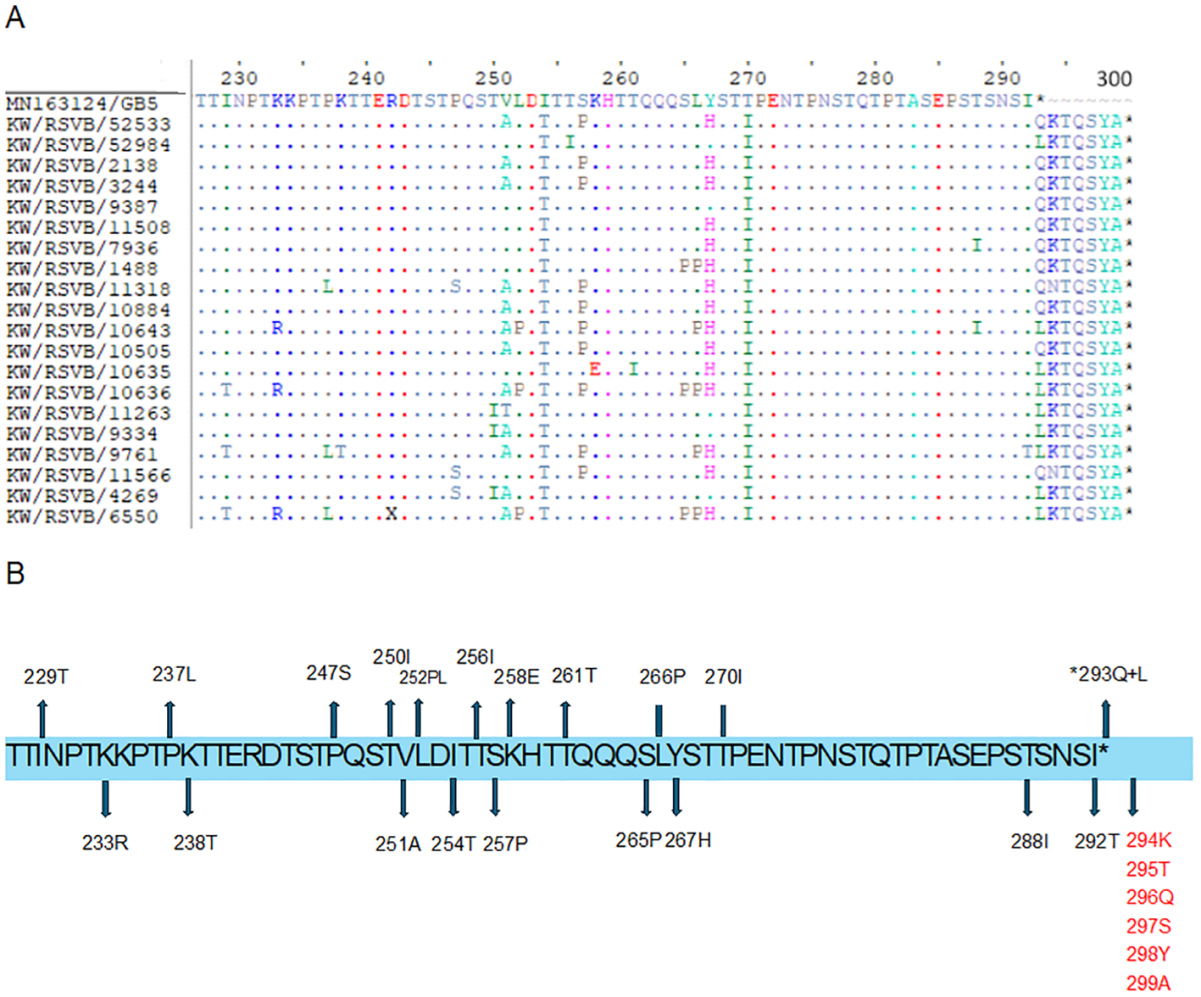
**(A)** Deduced amino acid alignment and mutations in the second hyper-variable region of the G protein (227-299) of RSV-B genotype. The Kuwaiti strains were aligned with the MN163124/GB5 prototype strain. Dots display identical residues. Stop codons are indicated by asterisks. **(B)** mutations in the amino acids of the Kuwaiti strains. An asterisk shows the stop codon in the prototype strain. Amino acids in red are inserted AA in the Kuwaiti strains.

Detailed analysis of the G protein second hyper-variable region of the Kuwaiti RSV-A strains revealed a total of 26 unique mutations as compared to the prototype strain, including the 11 high-frequency mutations that were considered molecular markers, as mentioned earlier (E232G, T253K, L274P, L286P, S289P, P290S, S292P, S293P, T296P, K297R, and the stop codon 297Q). Among these mutations, all RSV-A strains had L286P, S289P, K297R, and an insertion of CAG at the stop codon nucleotide resulted in an extension of the G protein by 1 AA (**Figures 4A, B**). In contrast, 20 unique nucleotide mutations were detected in the Kuwaiti RSV-B strains compared to the prototype strain, including the 13 high-frequency mutations considered molecular markers (I254T, T270I, stop codon 293Q/L, 294K/N (insertion), 295T (insertion), 296Q (insertion), 297S (insertion), 298Y (insertion), and 299A (insertion). Thus, there was an insertion of 21 coding nucleotides (AAAACCCAGTCATATGCTTAG) at the stop codon, resulting in an extension of the G protein of RSV-B strains by seven amino acids (QKTQSYA*) (**Figures 5A, B**).

## 4 Discussion

RSV is the leading cause of ARTI in children worldwide and an important pathogen in elderly and immunocompromised patients (Shi et al., 2017; Troeger et al., 2018). This study comprehensively investigated the molecular characterization of the G glycoproteins of RSV-A and B genotypes circulated in Kuwait during the 2020 and 2022 seasons using Nanopore sequencing technology to sequence the full-length gene of G protein. In this study, most of the RSV-positive patients were younger than 12 months old, and the median age of the patients was less than one year old. These results agreed with other studies (Glezen, 1986; Huang et al., 2024). We sequenced 84 full-length G genes, and our findings demonstrated that the RSV-A genotype (76%) was the predominant subtype, while the RSV-B genotype was detected in 24% of the RSV isolates. These findings support previous reports (Parveen et al., 2006; Zhang et al., 2010). Furthermore, according to Nextstrain designation, sub-genotype GA2.3.5 of RSV-A appeared to be predominated (59%), while sub-genotype GA2.3.3 was detected in 41% of the RSV-A isolates. On the other hand, all RSV-B genotypes were classified as GB5.0.5a sub-genotype. Phylogenetic analysis of the full-length G protein using different reference strains demonstrated that the GA2.3.5 sub-genotypes of RSV-A were divided into two main lineages and one of these lineages was divided further into two sub-lineages. The proposed sub-genotypes could be a novel addition to the diversity of RSV-A. This is supported by a nucleotide divergence of 0.07 and nucleotide identity of 93%, measured between these sub-genotypes and the reference strains. It’s important to note that some of the RSV-A strains were classified as GA2.3.3 according to the Nextstrain platform. However, our findings revealed that the nucleotide divergence between these RSV-A strains and the GA2.3.3 reference strain was 6.75, indicating that these strains are not closely related to the GA2.3.3 sub-genotypes. Furthermore, the GB5.0.5a sub-genotypes of RSV-B were further classified into two new and distinct sub-lineages. These findings suggest the emergence of new lineages and sub-lineages of GA2.3.5 and GB5.0.5a in Kuwait between 2020 and 2022. Further ongoing surveillance is necessary to confirm the presence of these new strains.

Because of immune pressure and the lack of proofreading capabilities of the RSV RNA-dependent polymerase, the virus can evolve and diversify over time. This evolution is imitated in the diversities of the F and G proteins. As a result, the RSV genotypes have changed over the past decades, and new genotypes have replaced the old ones (Schobel et al., 2016). Diversification of the circulated GA2.3.5 sub-genotype of RSV-A and GB5.0.5a sub-genotypes of RSV-B in Kuwait was well documented. Our data showed that, in both genotypes, the most variable region of the G protein was the second hypervariable region, although this region was less variable in the GB5.0.5a sub-genotypes. In addition, our data showed that the CX3C motif and heparin-binding domain (HBD) regions were conserved in both genotypes. The G protein binds to the CX3C chemokine receptor, CX3CR1, through the CX3C motif, thus facilitating the infection of primary human airway epithelial cells. Therefore, anti-G antibodies effectively neutralize RSV in these cells (Chirkova et al., 2015; Anderson et al., 2021). In addition, HBD mediates viral infection, where RSV binds to glycosaminoglycans (GAGs) on the cell surface (Feldman et al., 1999). Unlike our results, other studies showed that HBD may vary among different strains of RSV (Teng et al., 2001; Shields et al., 2003). Therefore, variability of RSV is believed to impact viral antigenic properties, evading the host immune response and facilitating viral infection, enabling the virus to spread competently or conferring a fitness advantage during circulation (van Niekerk and Venter, 2011; Duvvuri et al., 2015).

A mutational analysis was conducted on the G protein of RSV-A and RSV-B genotypes, revealing various mutations, including insertions, deletions, and stop codon mutations. Our study results indicated that the G proteins of RSV-A and RSV-B exhibit various unique mutations, with RSV-A having more mutations than RSV-B. Most of these mutations are situated in the second hypervariable region. In addition, 12 and 13 unique molecular markers were identified in RSV-A and RSV-B, respectively, compared to the prototype strains, which are located in the hypervariable region of the G protein, that possibly affect the antigenicity of the virus and can be used in the designation of the RSV strain. However, we cannot underestimate the importance of other amino acid changes that appear at lower percentages. In the intracellular cytoplasmic tail region, mutation T4N was detected in 59% of RSV-A, and mutation N4H was detected in 50% of RSV-B. Additionally, the K32R mutation was present in 30% of RSV-B genotypes. Although this region is not exposed to the cell surface, it contains B cell epitopes and mutations in this area can impact the antigenic characteristics of the G protein. Experimental evidence has shown that mutations L33P and L35P in RSV-A prevented the virus from interacting with neutralizing monoclonal antibodies. In the transmembrane region, the amino acid substitution I38V was detected in 27% of RSV-A genotypes. While there is limited information available on the variability of this region, changes in this area are believed to decrease the affinity of RSV-specific neutralizing antibodies. In the first hypervariable region, our data revealed the presence of 12 mutations in RSV-A genotypes compared to the prototype. In RSV-B genotypes, the data showed the presence of five mutations and three amino acid insertions (aa 158-160; KPK) that were conserved among all the strains. The first hypervariable region contains B cell epitopes (aa 66–90, 90–110, 129–152) and CD4+ T cell epitopes (aa 104–118). Therefore, mutations in these positions may alter the virus’s antigenic site. The central conserved domain of the G protein is highly conserved among strains because it is involved in the interaction with host target cells; however, our study documented the presence of N178G in 31% of RSV-A genotypes, and therefore, this substitution may interfere with this function. The results show that the RSV-A and RSV-B genotypes in Kuwait have distinct mutations and molecular markers that are not seen in other strains. This suggests the emergence of new virus lineages in Kuwait. This information can be used to develop more effective diagnostic and treatment strategies for RSV infections.

N- and O-glycosylation are important in maintaining the integrity of G protein and hence affect the antigenicity and virulence of RSV (García-Beato et al., 1996; Leemans et al., 2018).Our study’s results demonstrated high variability of N-glycosylation sites; the O-glycosylation sites were also intensively distributed along the G protein. We believe that the higher glycans content in the G protein may help RSV evade the host’s immune response by hindering the effective presentation of the G protein to immune cells (Melero et al., 2017; Krivitskaya et al., 2021). However, we need specific biochemical analyses to support these hypotheses.

The second hypervariable region is the most inconsistent domain of the G protein. In our study, RSV-A and RSV-B genotypes had 26 and 20 variable positions, respectively (**Figures 4**, **5**). Studies demonstrated that aa 229–240, 236–298, 265–273, 283–291 are B cell immunodominant determinants in humans, and therefore, any change in these positions can affect the anti-RSV immune response (Cane, 1997; Fuentes et al., 2016). Some of the mutations in RSV-A genotypes (248I, 274P, 290S, and 298Q) were previously shown to be positively selected in ON1/GA2 viruses (Agoti et al., 2014; Duvvuri et al., 2015; Esposito et al., 2015). This indicates that they are important for developing immunity during infection, and their variability may be influenced by host immune pressure. It’s important to note that an additional AA extended the stop codon in RSV-A genotypes, while seven additional AAs extended the stop codon in RSV-B genotypes. These insertions resulted in an increased length of the G protein.

## 5 Conclusion

The data from this study shows that the main strain of RSV circulating in Kuwait from 2020 to 2022 was RSV-A. Analysis of the full-length G protein sequence of RSV-A and RSV-B genotypes showed that the isolates belonged to sub-genotypes GA2.3.5 and GB5.0.5.a but formed new lineages. Many mutations and insertions were found in the antigenic epitopes of the G protein, along with high variability of N- and O-glycosylation sites. These changes may result from immune pressure and could impact the antigenic properties of currently circulating strains, potentially leading to new RSV variants capable of evading the immune response. This detailed study on the G protein of RSV-A and RSV-B could help in the development of more effective treatments and vaccines.

## Data Availability

The datasets presented in this study can be found in online repositories. The names of the repository/repositories and accession number(s) can be found in the article/**Supplementary Material**.

## References

[B1] Agoti C. N. Otieno J. R. Gitahi C. W. Cane P. A. Nokes D. J. (2014). Rapid spread and diversification of respiratory syncytial virus genotype ON1, Kenya. Emerg. Infect. Dis. 20, 950–959. doi: 10.3201/eid2006.131438 24856417 PMC4036793

[B2] Ahmed A. Haider S. H. Parveen S. Arshad M. Alsenaidy H. A. Baaboud A. O.. (2016). Co-circulation of 72bp duplication group A and 60bp duplication group B respiratory syncytial virus (RSV) strains in Riyadh, Saudi Arabia during 2014. PLoS One. 11, e0166145. doi: 10.1371/journal.pone.0166145 27835664 PMC5106011

[B3] Anderson L. Jadhao S. Paden C. Tong S. (2021). Functional features of the respiratory syncytial virus G protein. Viruses. 13, 1214. doi: 10.3390/v13071214 34372490 PMC8310105

[B4] Cane P. A. (1997). Analysis of linear epitopes recognised by the primary human antibody response to a variable region of the attachment (G) protein of respiratory syncytial virus. J. Med. Virol. 51, 297–304. doi: 10.1002/(SICI)1096-9071(199704)51:4<297::AID-JMV7>3.0.CO;2-0 9093944

[B5] Chirkova T. Lin S. Oomens A. G. P. Gaston K. A. Boyoglu-Barnum S. Meng J.. (2015). CX3CR1 is an important surface molecule for respiratory syncytial virus infection in human airway epithelial cells. J. Gen. Virol. 96, 2543–2556. doi: 10.1099/vir.0.000218 26297201 PMC4635495

[B6] Duvvuri V. R. Granados A. Rosenfeld P. Bahl J. Eshaghi A. Gubbay J. B. (2015). Genetic diversity and evolutionary insights of respiratory syncytial virus A ON1 genotype: global and local transmission dynamics. Sci. Rep. 5, 14268. doi: 10.1038/srep14268 26420660 PMC4588507

[B7] Esposito S. Piralla A. Zampiero A. Bianchini S. Di Pietro G. Scala A.. (2015). Characteristics and their clinical relevance of respiratory syncytial virus types and genotypes circulating in northern Italy in five consecutive winter seasons. PLoS One. 10, e0129369. doi: 10.1371/journal.pone.0129369 26047100 PMC4457818

[B8] Feldman S. A. Hendry R. M. Beeler J. A. (1999). Identification of a linear heparin binding domain for human respiratory syncytial virus attachment glycoprotein G. J. Virol. 73, 6610–6617. doi: 10.1128/JVI.73.8.6610-6617.1999 10400758 PMC112745

[B9] Fuentes S. Coyle E. M. Beeler J. Golding H. Khurana S. (2016). Antigenic fingerprinting following primary RSV infection in young children identifies novel antigenic sites and reveals unlinked evolution of human antibody repertoires to fusion and attachment glycoproteins. PloS Pathog. 12, e1005554. doi: 10.1371/journal.ppat.1005554 27100289 PMC4839671

[B10] García-Beato R. Martínez I. Francí C. Real F. X. García-Barreno B. Melero J. A. (1996). Host cell effect upon glycosylation and antigenicity of human respiratory syncytial virus G glycoprotein. Virology. 221, 301–309. doi: 10.1006/viro.1996.0379 8661440

[B11] Gimferrer L. Campins M. Codina M. G. Martín M. del C. Fuentes F.. (2015). Molecular epidemiology and molecular characterization of respiratory syncytial viruses at a tertiary care university hospital in Catalonia (Spain) during the 2013–2014 season. J. Clin. Virol. 66, 27–32. doi: 10.1016/j.jcv.2015.02.018 25866332

[B12] Glezen W. P. (1986). Risk of primary infection and reinfection with respiratory syncytial virus. Arch. Pediatr. Adolesc. Med. 140, 543. doi: 10.1001/archpedi.1986.02140200053026 3706232

[B13] Goya S. Galiano M. Nauwelaers I. Trento A. Openshaw P. J. Mistchenko A. S.. (2020). Toward unified molecular surveillance of RSV: a proposal for genotype definition. Influenza Other Respir Viruses. 14, 274–285. doi: 10.1111/irv.12715462 32022426 PMC7182609

[B14] Hadfield J. Megill C. Bell S. M. Huddleston J. Potter B. Callender C.. (2018). Nextstrain: real-time tracking of pathogen evolution. Bioinformatics. 34, 4121–4123. doi: 10.1093/bioinformatics/bty407 29790939 PMC6247931

[B15] Hall C. B. Simőes E. A. F. Anderson L. J. (2013). Clinical and epidemiologic features of respiratory syncytial virus. 372, 39–57. doi: 10.1007/978-3-642-38919-1_2 24362683

[B16] Huang L. Xu Y. Yang Y. Dong H. Luo Q. Chen Z.. (2024). Molecular epidemiology and clinical characteristics of respiratory syncytial virus in hospitalized children during winter 2021–2022 in Bengbu, China. Front. Public Health. 11. doi: 10.3389/fpubh.2023.1310293 PMC10791987 38235154

[B17] Leemans A. Boeren M. van der Gucht W. Pintelon I. Roose K. Schepens B.. (2018). Removal of the N-Glycosylation Sequon at Position N116 Located in p27 of the Respiratory Syncytial Virus Fusion Protein Elicits Enhanced Antibody Responses after DNA Immunization. Viruses. 10, 426. doi: 10.3390/v10080426 30110893 PMC6115940

[B18] Li Y. Wang X. Blau D. M. Caballero M. T. Feikin D. R. Gill C. J.. (2022). Global, regional, and national disease burden estimates of acute lower respiratory infections due to respiratory syncytial virus in children younger than 5 years in 2019: a systematic analysis. Lancet. 399, 2047–2064. doi: 10.1016/S0140-6736(22)00478-0 35598608 PMC7613574

[B19] Lu B. Liu H. Tabor D. E. Tovchigrechko A. Qi Y. Ruzin A.. (2019). Emergence of new antigenic epitopes in the glycoproteins of human respiratory syncytial virus collected from a US surveillance study 2015–17. Sci. Rep. 9, 3898. doi: 10.1038/s41598-019-40387-y 30846850 PMC6405860

[B20] Madi N. Chehadeh W. Asadzadeh M. Al-Turab M. Al-Adwani A. (2018). Analysis of genetic variability of respiratory syncytial virus groups A and B in Kuwait. Arch Virol. 163, 2405–2413. doi: 10.1007/s00705-018-3881-z 29777370 PMC7087269

[B21] McLellan J. S. Ray W. C. Peeples M. E. (2013). Structure and function of respiratory syncytial virus surface glycoproteins. 372, 83–104. doi: 10.1007/978-3-642-38919-1_4 PMC4211642 24362685

[B22] Parveen S. Sullender W. M. Fowler K. Lefkowitz E. J. Kapoor S. K. Broor S. (2006). Genetic variability in the G protein gene of group A and B respiratory syncytial viruses from India. J. Clin. Microbiol. 44, 3055–3064. doi: 10.1128/JCM.00187-06 16954227 PMC1594720

[B23] Peret T. C. T. Hall C. B. Hammond G. W. Piedra P. A. Storch G. A. Sullender W. M.. (2000). Circulation patterns of group A and B human respiratory syncytial virus genotypes in 5 communities in North America. J. Infect. Dis. 181, 1891–1896. doi: 10.1086/315508 10837167

[B24] Schobel S. A. Stucker K. M. Moore M. L. Anderson L. J. Larkin E. K. Shankar J.. (2016). Respiratory Syncytial Virus whole-genome sequencing identifies convergent evolution of sequence duplication in the C-terminus of the G gene. Sci. Rep. 6, 26311. doi: 10.1038/srep26311 27212633 PMC4876326

[B25] Shi T. Denouel A. Tietjen A. K. Campbell I. Moran E. Li X.. (2020). Global disease burden estimates of respiratory syncytial virus–associated acute respiratory infection in older adults in 2015: A systematic review and meta-analysis. J. Infect. Dis. 222, S577–S583. doi: 10.1093/infdis/jiz059 30880339

[B26] Shi T. McAllister D. A. O’Brien K. L. Simoes E. A. F. Madhi S. A. Gessner B. D.. (2017). Global, regional, and national disease burden estimates of acute lower respiratory infections due to respiratory syncytial virus in young children in 2015: a systematic review and modelling study. Lancet. 390, 946–958. doi: 10.1016/S0140-6736(17)30938-8 28689664 PMC5592248

[B27] Shields B. Mills J. Ghildyal R. Gooley P. Meanger J. (2003). Multiple heparin binding domains of respiratory syncytial virus G mediate binding to mammalian cells. Arch. Virol. 148, 1987–2003. doi: 10.1007/s00705-003-0139-0 14551820

[B28] Tamura S. K. Stecher G. Kumar S. (2021). MEGA11: Molecular Evolutionary Genetics Analysis Version 11. Mol. Biol. Evol. 38, 3022–3027. doi: 10.1093/molbev/msab120 33892491 PMC8233496

[B29] Teng M. N. Whitehead S. S. Collins P. L. (2001). Contribution of the respiratory syncytial virus G glycoprotein and its secreted and membrane-bound forms to virus replication *in vitro* and *in vivo* . Virology. 289, 283–296. doi: 10.1006/viro.2001.1138 11689051

[B30] Troeger C. Blacker B. Khalil I. A. Rao P. C. Cao J. Zimsen S. R. M.. (2018). Estimates of the global, regional, and national morbidity, mortality, and aetiologies of lower respiratory infections in 195 countries 1990–2016: a systematic analysis for the Global Burden of Disease Study 2016. Lancet Infect. Dis. 18, 1191–1210. doi: 10.1016/S1473-3099(18)30310-4 30243584 PMC6202443

[B31] van Niekerk S. Venter M. (2011). Replacement of previously circulating respiratory syncytial virus subtype B strains with the BA genotype in South Africa. J. Virol. 85, 8789–8797. doi: 10.1128/JVI.02623-10 21715483 PMC3165815

[B32] Widjojoatmodjo M. N. Boes J. van Bers M. van Remmerden Y. Roholl P. J. Luytjes W. (2010). A highly attenuated recombinant human respiratory syncytial virus lacking the G protein induces long-lasting protection in cotton rats. Virol. J. 7, 114. doi: 10.1186/1743-422X-7-114 20525213 PMC2887800

[B33] Zhang Z. Du L. Chen X. Zhao Y. Liu E. Yang X.. (2010). Genetic variability of respiratory syncytial viruses (RSV) prevalent in southwestern China from 2006 to 2009: emergence of subgroup B and A RSV as dominant strains. J. Clin. Microbiol. 48, 1201–1207. doi: 10.1128/JCM.02258-09 20147636 PMC2849604

