# Supplementary material for: Circulation of new lineages of RSV-A and RSV-B in Kuwait shows high diversity in the N- and O-linked glycosylation sites in the G protein between 2020 and 2022
Source: Front Cell Infect Microbiol. 2024 Aug 16;14:1445115. doi: 10.3389/fcimb.2024.1445115 (PMC11362131; doi:10.3389/fcimb.2024.1445115)
Supplement: Supplementary file 1 [file Table1.docx]

**Supplement**

**S1 Table: List of Kuwaiti RSV-A and RSV-B strains used in this study (N=84).**

| **GenBank Accession No.** | **Strain name** | **Genotype** |
| --- | --- | --- |
| PP151342 | KW/RSVA/46550 | GA2.3.5 |
| PP151343 | KW/RSVA/45535 | GA2.3.5 |
| PP151344 | KW/RSVA/52942 | GA2.3.5 |
| PP151345 | KW/RSVA/52939 | GA2.3.5 |
| PP151346 | KW/RSVA/52941 | GA2.3.5 |
| PP151347 | KW/RSVA/1750 | GA2.3.5 |
| PP151348 | KW/RSVA/3657 | GA2.3.5 |
| PP151349 | KW/RSVA/1765 | GA2.3.5 |
| PP151350 | KW/RSVA/344 | GA2.3.5 |
| PP151351 | KW/RSVA/52940 | GA2.3.5 |
| PP151352 | KW/RSVA/11509 | GA2.3.5 |
| PP151353 | KW/RSVA/46102 | GA2.3.5 |
| PP151354 | KW/RSV/52155 | GA2.3.5 |
| PP151355 | KW/RSVA/44240 | GA2.3.5 |
| PP151356 | KW/RSVA/45688 | GA2.3.5 |
| PP151357 | KW/RSVA/52206 | GA2.3.5 |
| PP151358 | KW/RSVA/52534 | GA2.3.5 |
| PP151359 | KW/RSVA/52536 | GA2.3.5 |
| PP151360 | KW/RSVA/52740 | GA2.3.5 |
| PP151361 | KW/RSVA/52923 | GA2.3.5 |
| PP151362 | KW/RSVA/53056 | GA2.3.5 |
| PP151363 | KW/RSVA/53109 | GA2.3.5 |
| PP151364 | KW/RSVA/40350 | GA2.3.5 |
| PP151365 | KW/RSVA/46811 | GA2.3.5 |
| PP151366 | KW/RSVA/46919 | GA2.3.5 |
| PP151367 | KW/RSVA/5278 | GA2.3.5 |
| PP151368 | KW/RSVA/5738 | GA2.3.5 |
| PP151369 | KW/RSVA/43617 | GA2.3.5 |
| PP151370 | KW/RSVA/50956 | GA2.3.5 |
| PP151371 | KW/RSVA/43722 | GA2.3.5 |
| PP151372 | KW/RSVA/46963 | GA2.3.5 |
| PP151373 | KW/RSVA/2253 | GA2.3.5 |
| PP151374 | KW/RSVA/2187 | GA2.3.5 |
| PP151375 | KW/RSVA/47618 | GA2.3.5 |
| PP151376 | KW/RSVA/39616 | GA2.3.5 |
| PP151377 | KW/RSVA/48158 | GA2.3.5 |
| PP151378 | KW/RSVA/46733 | GA2.3.5 |
| PP151379 | KW/RSVA/46414 | GA2.3.5 |
| PP151380 | KW/RSVA/43736 | GA2.3.5 |
| PP151381 | KW/RSVA/45534 | GA2.3.5 |
| PP151382 | KW/RSVA/515 | GA2.3.5 |
| PP151383 | KW/RSVA/51 | GA2.3.5 |
| PP151384 | KW/RSVA/45423 | GA2.3.5 |
| PP151385 | KW/RSVA/45186 | GA2.3.5 |
| PP151386 | KW/RSVA/45178 | GA2.3.5 |
| PP151387 | KW/RSVA/44695 | GA2.3.5 |
| PP151388 | KW/RSVA/44512 | GA2.3.5 |
| PP151389 | KW/RSVA/44196 | GA2.3.5 |
| PP151390 | KW/RSVA/44163 | GA2.3.5 |
| PP151391 | KW/RSVA/41556 | GA2.3.5 |
| PP151392 | KW/RSVA/40839 | GA2.3.5 |
| PP151393 | KW/RSVA/40449 | GA2.3.5 |
| PP151394 | KW/RSVA/40419 | GA2.3.5 |
| PP151395 | KW/RSVA/36(B) | GA2.3.5 |
| PP151396 | KW/RSVA/36 | GA2.3.5 |
| PP151397 | KW/RSVA/49662 | GA2.3.5 |
| PP151398 | KW/RSVA/49525 | GA2.3.5 |
| PP151399 | KW/RSVA/48464 | GA2.3.5 |
| PP151400 | KW/RSVA/15 | GA2.3.5 |
| PP151401 | KW/RSVA/49559 | GA2.3.5 |
| PP151402 | KW/RSVA/11262 | GA2.3.5 |
| PP151403 | KW/RSVA/0283 | GA2.3.5 |
| PP151404 | KW/RSVA/45387 | GA2.3.5 |
| PP151405 | KW/RSVA/22 | GA2.3.5 |
| PP135042 | KW/RSVB/52533 | GB5.0.5a |
| PP135043 | KW/RSVB/52984 | GB5.0.5a |
| PP135044 | KW/RSVB/2138 | GB5.0.5a |
| PP135045 | KW/RSVB/3244 | GB5.0.5a |
| PP135046 | KW/RSVB/9387 | GB5.0.5a |
| PP135047 | KW/RSVB/11508 | GB5.0.5a |
| PP135048 | KW/RSVB/7936 | GB5.0.5a |
| PP135049 | KW/RSVB/1488 | GB5.0.5a |
| PP135050 | KW/RSVB/11318 | GB5.0.5a |
| PP135051 | KW/RSVB/10884 | GB5.0.5a |
| PP135052 | KW/RSVB/10643 | GB5.0.5a |
| PP135053 | KW/RSVB/10505 | GB5.0.5a |
| PP135054 | KW/RSVB/10635 | GB5.0.5a |
| PP135055 | KW/RSVB/10636 | GB5.0.5a |
| PP135056 | KW/RSVB/11263 | GB5.0.5a |
| PP135057 | KW/RSVB/9334 | GB5.0.5a |
| PP135058 | KW/RSVB/9761 | GB5.0.5a |
| PP135059 | KW/RSVB/11566 | GB5.0.5a |
| PP135060 | KW/RSVB/4269 | GB5.0.5a |
| PP135061 | KW/RSVB/6550 | GB5.0.5a |

**S2 Table: List of all amino acid changes in the G protein of RS-A and RSV-B circulated in Kuwait.**

| **RSV-A** | **RSV-B** |
| --- | --- |
| T4N | N4H |
| R8S | K32R |
| R15K | S100G |
| I38V | P105L |
| L71P | S119P |
| T80M | A131T |
| I99T | T137I |
| S100N | 158K^♥^ |
| F101/S | 159P^♥^ |
| T113I | 160K |
| L115P | T200I |
| I118T | N204K |
| T119P | P207T |
| T129S | P216S |
| V131D | P219L |
| K134E | P223T |
| R151H | K224E |
| N178G | I229T |
| R204N | K233R |
| P206Q | P237L |
| K209R | K238T |
| T210A | P247S |
| P215L | T250I |
| V225A | V251A |
| L226P | L252P |
| P230T | I254T |
| E232G | T256I |
| K233E | S257P |
| R244I | K258E |
| T245I | T261I |
| L248I | S265P |
| T253K | L260P |
| G254R | Y267H |
| H258Q/L | T270I |
| E262M/K | T288I |
| L265F | I292T |
| H266L | 293Q/L |
| T269S | 294K^♥^ |
| S270P | 995T^♥^ |
| Y273H/N | 296Q^♥^ |
| L274P | 297S^♥^ |
| P276Q | 298Y^♥^ |
| Y280S | 299A^♥^ |
| L286P |  |
| S289P |  |
| P290S |  |
| S292P |  |
| S293P |  |
| T296P |  |
| K297R |  |
| *298Q |  |

*Stop codon

^♥^ AA insertion
